# Utilitarianism in minimal-group decision making is less common than equality-based morality, mostly harm-oriented, and rarely impartial

**DOI:** 10.1038/s41598-020-70199-4

**Published:** 2020-08-07

**Authors:** Arne Roets, Dries H. Bostyn, Jonas De keersmaecker, Tessa Haesevoets, Jasper Van Assche, Alain Van Hiel

**Affiliations:** Department of Developmental, Personality, and Social Psychology, Ghent University, Ghent, Belgium

**Keywords:** Psychology, Human behaviour

## Abstract

In the study of utilitarian morality, the sacrificial dilemma paradigm has been the dominant approach for years. However, to address some of the most pressing issues in the current research literature, the present studies adopt an alternative approach by using a minimal group paradigm in which participants have to make decisions about the allocation of resources. This approach allows not only to pit utilitarianism against equality-based morality, but also to study these modes of morality for both harm and benefit, and to directly address the role of group identity affecting the (im)partial nature of ‘utilitarian’ (i.e., outcome maximizing) decisions. In our experiments, across four different samples (total N = 946), we demonstrate that although participants generally prefer equality-based allocations over maximizing distributions, outcome maximizing choices become more prevalent when they served to minimize harm compared to maximizing benefit. Furthermore, reducing the objective value of the equal distribution outcomes further prompts participants to adopt a more utilitarian approach in situations involving harm, but has little effect in situations where benefits have to be distributed. Finally, the introduction of (minimal) group identity consistently demonstrates that decisions that maximize the overall outcome are more likely if they also serve the ingroup compared to when they rather serve the outgroup. We discuss how these findings have meaningful implications that may be especially relevant for recent movements that advocate a utilitarian approach to charity, and for our understanding of (im)partiality in lay people’s ‘utilitarian’ decision making.

## Introduction

Over the past 2 decades, ‘sacrificial dilemmas’ (e.g., the trolley dilemma and the footbridge dilemma)^1^ have become the cornerstone and primary methodology in the surging research literature on utilitarian moral decision making. In their archetypical form, these dilemmas present participants with a hypothetical situation that asks them to decide whether they would sacrifice the life of one anonymous person to save the lives of multiple others. For example: the prototypical dilemma asks participants to imagine a runaway trolley train headed for a deadly collision with five unsuspecting workmen. While those five men could be saved by diverting the runaway train to a second track, doing so would cause a deadly collision with a single workman. People who decide to sacrifice the one workman to save the lives of the five others are considered to display utilitarian morality, whereas those who reject the option to sacrifice a life as an acceptable trade-off, display non-utilitarian or deontological morality^1^. This quite straightforward approach and empirical operationalization of a philosophical construct as intricate as utilitarianism has given an invaluable boost to empirical research in moral psychology, leading to a number of high-profile studies (e.g.,^2–5^).

However, recently, critical voices have argued that research on utilitarian vs non-utilitarian modes of morality relying on sacrificial dilemmas, can only provide a partial picture of utilitarian morality. Most notably, it has been asserted that the use of such sacrificial dilemmas as the primary or sole means to investigate utilitarianism “ignores the positive, altruistic core of utilitarianism, which is characterized by impartial concern for the well-being of everyone” (^6^, p. 131). This work (see also,^7,8^) as such highlights two important aspects of utilitarianism that are not considered by the traditional sacrificial dilemma approach: (1) the importance of (also) focusing on maximizing benefit as a key aspect of utilitarianism (rather than only looking at minimizing harm), and (2) the requirement of impartiality of decisions in classic utilitarianism.

### Minimizing harm versus maximizing benefit

The extent to which instrumental harm (i.e., doing harm for the greater good) is central to utilitarian morality, as well as whether or not decisions to minimize harm in sacrificial dilemmas should be considered as directly indicative of utilitarian reasoning, has been the subject of debate in recent years (e.g.,^8,9^). However, regardless of one’s position in this debate, it can hardly be contested that research that simultaneously focusses on both minimizing harm *and* on maximizing benefit can provide a more encompassing picture of utilitarian decision making. But equally important, it may also shed light on possible, fundamental differences in (utilitarian) moral reasoning about harm and benefit in lay people. For example, it has recently been shown that priming intuition versus deliberation decreases utilitarian responses when involving instrumental harm, but not when involving impartial beneficence^10^. Previous research in various domains has also shown that negative events tend to elicit stronger and different psychological reactions compared to positive events (e.g.,^11,12^), and research on morality in particular has suggested different evaluation standards of blame and praise when we judge the morality of negative versus positive actions^13–15^. Furthermore, it has been argued that moral cognition seems inherently more attuned to evaluating harmful compared to beneficial actions^16^, and that people are especially wary about causing harm to others (and often even prefer receiving pain themselves rather than to inflict it on others)^17^.

These various findings may be directly relevant for people’s use of utilitarianism-based morality in harm versus benefit situations. In particular, we hypothesize that in situations that involve inflicting harm, people are especially motivated to minimize such harms and hence should be more inclined to follow a utilitarian reasoning to that end, whereas in situations where benefits are distributed, there may be less pressing concerns to maximize benefit and therefore more room for other moral principles (i.e., equality, see below).

### Impartiality

Impartiality is deemed central to utilitarian morality. In particular, it has been argued^7^ that utilitarianism dictates us to approach moral decisions as if ‘from the point of view of the universe’^18^, and to completely disregard any particular loyalty or preferential treatment to ourselves, those close to us, or those who belong to our own group. To be truly utilitarian, decisions should only be concerned with maximizing overall benefit (or minimizing overall harm), without regard for who the individual targets are. For example, truly impartial utilitarian judgments make no distinction between options that are relatively more favorable for an outgroup versus options that are relatively more favorable to an ingroup, insofar the overall outcome is the same. However, although some studies have considered the role of the identity of the targets (e.g.,^19,20^), this issue remains unaddressed in most research using the traditional sacrificial dilemma approach, simply because these hypothetical dilemmas usually contain no information whatsoever that could lead to partiality. Or as researchers recently have put it: the current state of the literature is that of the study of ‘The moral psychology of raceless, genderless strangers’^21^. These authors therefore argue “for the importance of incorporating identity into moral psychology” (p. 216).

### The present study

Based on the current state of the literature on the psychological study of utilitarian morality, and on the recent critiques and calls to expand the field, we aimed to develop a series of studies that can advance our understanding by going beyond the traditional approach of sacrificial dilemmas. To do so, we based our studies on the seminal work by Tajfel^22^ on the minimal group paradigm. Using (an adapted version of) this methodology as a starting point to study utilitarianism has considerable advantages. First, the resource allocation paradigm is very suitable to study whether and when people use utilitarian principles in a context that is less hypothetical, and certainly less extreme, compared to traditional sacrificial dilemmas. Indeed, how much we can (expect to) learn about real-life moral decision making from research using this type of extreme sacrificial dilemmas, has been an ongoing concern exactly because of the hypothetical and unrealistic nature of these dilemmas (see e.g.,^23,24^). Secondly, the allocation matrices from which participants have to pick an option, provide a straightforward opportunity to study utilitarian decision making both in situations involving benefit (e.g. bonus points) and in situations involving harm (e.g., penalties) simultaneously, and responses to both can be directly compared in a within-subjects design. Thirdly, developed to investigate ingroup favoritism, the minimal groups paradigm inherently incorporates group identity as a core variable in the design, thereby making it especially suitable to study the (im)partiality of people’s decision making (see e.g.,^25^). Finally, whereas in traditional sacrificial dilemmas, a prohibiting deontological morality is the only alternative for utilitarianism, the minimal group paradigm allows to pit utilitarian morality against a different (and arguably more commonly used and relatable) moral principle: equality. Recent work in the context of the Moral Machine Experiment^26^ suggests that, if only given the option to do so, people often prefer to treat people equally^27^, and the extensive fairness literature (e.g.,^28–31^) has demonstrated the value people attach to equal outcomes in resource allocations. However, being concerned about equality is distinctively non-utilitarian, because although in some cases it may be framed as a form of consequentialism, it does not aim for the greater overall benefit^7^.

## Results

We report statistical tests based on linear models because the results from this approach are straightforward and easy to interpret. Results based on an alternative, multinomial approach are available through the osf page associated with this project (https://osf.io/mbcqp/). Importantly, all major findings and conclusions are supported by both analytical methods.

*Study 1 *data were collected in two independent samples (*N* = 217, and *N* = 208). Participants first had to choose which of two paintings (Klee or Kandinsky) they liked best, after which they received a task in which they had to allocate points to pairs of other students, identified only by their participant code and painting preference group. The allocations were made through a series of matrices modelled as simplified versions of Tajfel’s matrices, which presented either a distribution of bonus points, or of penalties. For each matrix (e.g., + 4/+ 4 vs + 6/+ 4 vs + 7/+ 2), participants had to choose one of three distribution options: the ‘Equality’ option simply distributed points equally between the two targets (e.g., + 4/+ 4). The ‘Maximizing’ option presented a distribution that awarded the most points combined, hence maximizing overall outcome and thereby aligning with utilitarian morality. However, points were not equally distributed between the two recipients (e.g. + 6/+ 4). Note that the presence of this option also entails that the matrices are not a zero-sum situation where relative gains for one party inevitably result in an equally large relative loss for the other party. Importantly, in half of the matrices the maximizing option was relatively more favorable for the ingroup member compared to the outgroup member, whereas in the other half of the matrices it was relatively more favorable for the outgroup member. As such, the present design allows to investigate whether the ‘utilitarian’, outcome maximizing approach, is used in an impartial way. Finally, the ‘Competition’ option provided the most absolute and relative benefit for the fellow ingroup member at the expense of the outgroup member (e.g., + 7/+ 2). Insofar this option can be linked to morality, it certainly demonstrates uncut partiality.

### General distribution of equality, maximizing, and competition choices (Study 1)

The distribution of choices across the benefit vs harm, and ingroup vs outgroup maximizing advantage matrices in Sample 1 and Sample 2 show that, in both samples, the Equality option (*M* = 0.75, *SE* = 0.02, and *M* = 0.71, *SE* = 0.02) was chosen most frequently, and significantly more than the Maximizing option (*M* = 0.16, *SE* = 0.02; *M* = 0.20, *SE* = 0.02): *t*(216) = 14.83, Cohen’s *d* = 1.90, and *t*(207) = 12.39, *d* = 1.65, both p < 0.001. The Competition option was chosen the least frequently (*M* = 0.09, *SE* = 0.02; *M* = 0.08, *SE* = 0.01), and significantly less often than the Maximizing option: *t*(216) =  − 3.17, *p* = 0.002, *d* = 0.30, and *t*(207) = − 5.28, *p* < 0.001, *d* = 0.51.

### Impact of outcome type and group benefit on maximizing choice (Study 1)

**Figure 1:**
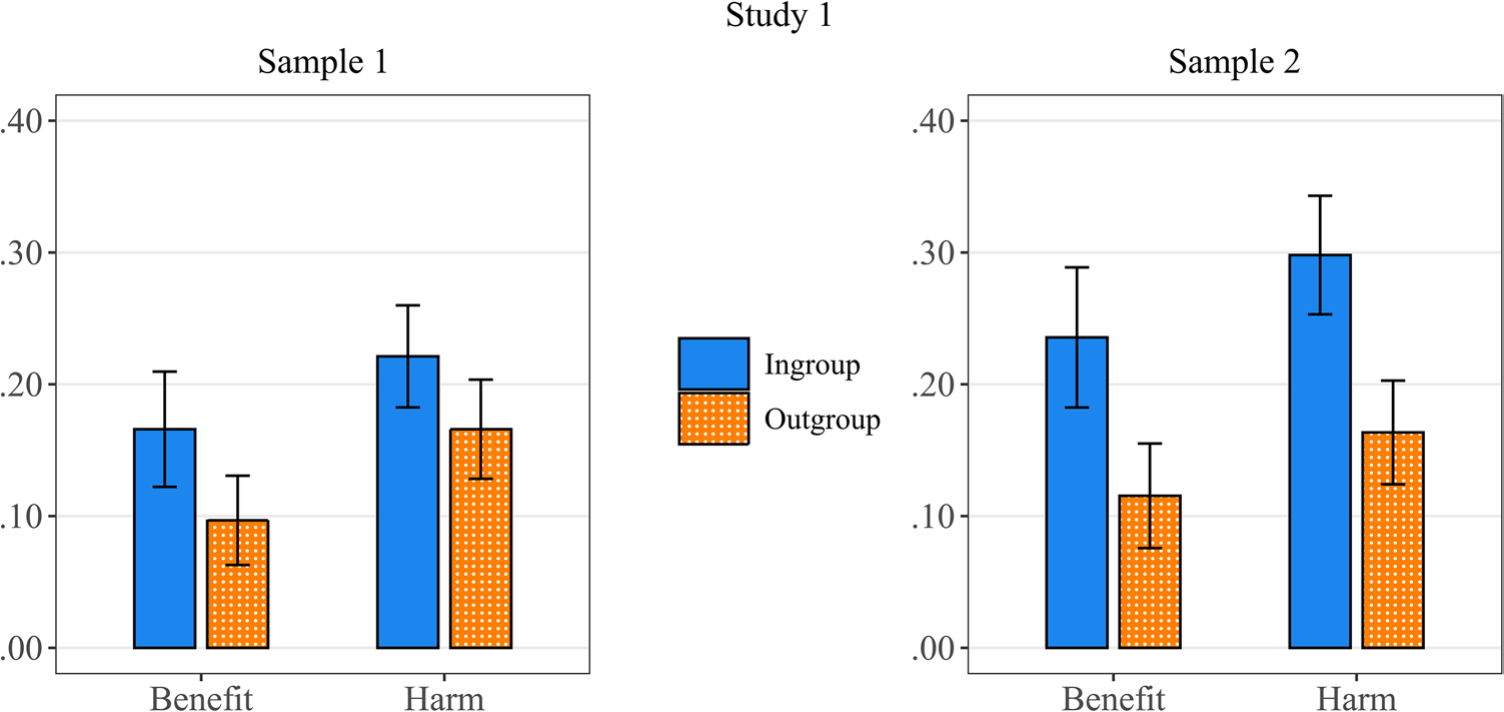
Proportion of outcome Maximizing choices as a function of target group (Ingroup versus Outgroup) and type of outcome (Benefit versus Harm). The error bars represent 95% confidence intervals.

We tested if outcome Maximizing choices are influenced by the type of outcome, i.e., whether participants had to distribute bonus points (benefit) or penalties (harm), and if Maximizing choices are influenced by the advantaged group, i.e., whether the choice was relatively more favorable to the ingroup member or to the outgroup member. Therefore, in each sample, a 2 × 2 Repeated measures ANOVA was conducted on the proportion of Maximizing choices, with type of outcome (Benefit vs Harm) and group (relative ingroup benefit vs relative outgroup benefit) as within-subject factors (see Fig. 1).

The significant main effect of Outcome type; *F*(1,216) = 7.75, *p* = 0.006, Cohen’s *f* = 0.24, and F(1,207) = 4.85, *p* = 0.029, *f* = 0.21, in sample 1 and sample 2 respectively, showed that participants chose the outcome Maximizing option significantly more when it served to minimize harm (*M* = 0.19, *SE* = 0.02, and *M* = 0.23, *SE* = 0.03), compared to when it maximized benefit (*M* = 0.13, *SE* = 0.02, and *M* = 0.18, *SE* = 0.02).

The significant main effect of beneficiary group; *F*(1,216) = 11.06, *p* = 0.001, *f* = 0.24, and *F*(1,207) = 28.67, *p* < 0.001, *f* = 0.48, in sample 1 and sample 2 respectively, demonstrated that participants were also more inclined to choose the Maximizing option when this option relatively benefited the ingroup member more (*M* = 0.19, *SE* = 0.02, and *M* = 0.27, *SE* = 0.03), compared to when it relatively benefited the outgroup member more (*M* = 0.13, *SE* = 0.02, and *M* = 0.14, *SE* = 0.02).

Finally, no significant interaction effect was found; *F*(1,216) = 0.16, *p* = 0.692, *f* = 0.03, and *F*(1,207) = 0.15, *p* = 0.697, *f* = 0.03, in sample 1 and sample 2 respectively (see Fig. 1).

*Study 2* data were collected in two new, independent samples (*N* = 268, and *N* = 253). Although the low overall proportions of Maximizing choices in Study 1 are informative of how (un)common such choices are when competing with Equality-based morality, they may hamper a powerful examination of how harm vs benefit, and outgroup vs ingroup may influence Maximizing choices. Therefore, in the first part of the second study (further referred to as Study 2a), we kept an identical design but reduced the objective value of the Equality option by lowering the bonus points or increasing the penalties in these choices. In essence, we adapted the matrices to ensure that the Equality options were the objectively worst choice out of all possible options in terms of overall and individual outcomes (e.g., + 6/+ 4 vs + 2/+ 2 vs + 7/+ 2, for the Maximizing, Equality, and Competition option respectively). Additionally, in the second part of this study (further referred to as Study 2b), we presented participants with an additional set of matrices that contained both stimuli with a standard value for the Equality option (as in Study 1) and with a low value for the Equality option (as in Study 2a). Unlike the previous set however, in these matrices, participants had to distribute the bonuses/penalties between either two ingroup members, or between two outgroup members. The latter (within-subject) manipulation allowed to also test whether participants are more inclined to maximize outcomes when distributing points within the ingroup compared to distributing points within the outgroup.

### General distribution of equality, maximizing, and competition choices between groups (Study 2a)

The distribution of choices across the benefit vs harm, and ingroup vs outgroup maximizing advantage matrices in Sample 3 and Sample 4 show a different pattern compared to the samples of Study 1. With the adapted matrices, the proportions representing how often the Equality option (*M* = 0.51, *SE* = 0.04, and *M* = 0.49, *SE* = 0.04) and the Utilitarian option (*M* = 0.42, *SE* = 0.03; *M* = 0.40, *SE* = 0.03) were chosen, were much closer, although the difference was still significant; *t*(267) = 2.268, *p* = 0.024, Cohen’s *d* = 0.27, and *t*(252) = 2.295, *p* = 0.023, *d* = 0.28, for sample 3 and sample 4, respectively. The Competition option was chosen the least frequently (*M* = 0.07, *SE* = 0.02; *M* = 0.10, *SE* = 0.02), and significantly less often than the Utilitarian option; *t*(267) =  − 13.458, *d* = − 1.25, and *t*(252) =  − 12.275, *d* = − 1.11, both *p* < 0.001.

### Impact of outcome type and group benefit on maximizing choice (Study 2a)

**Figure 2:**
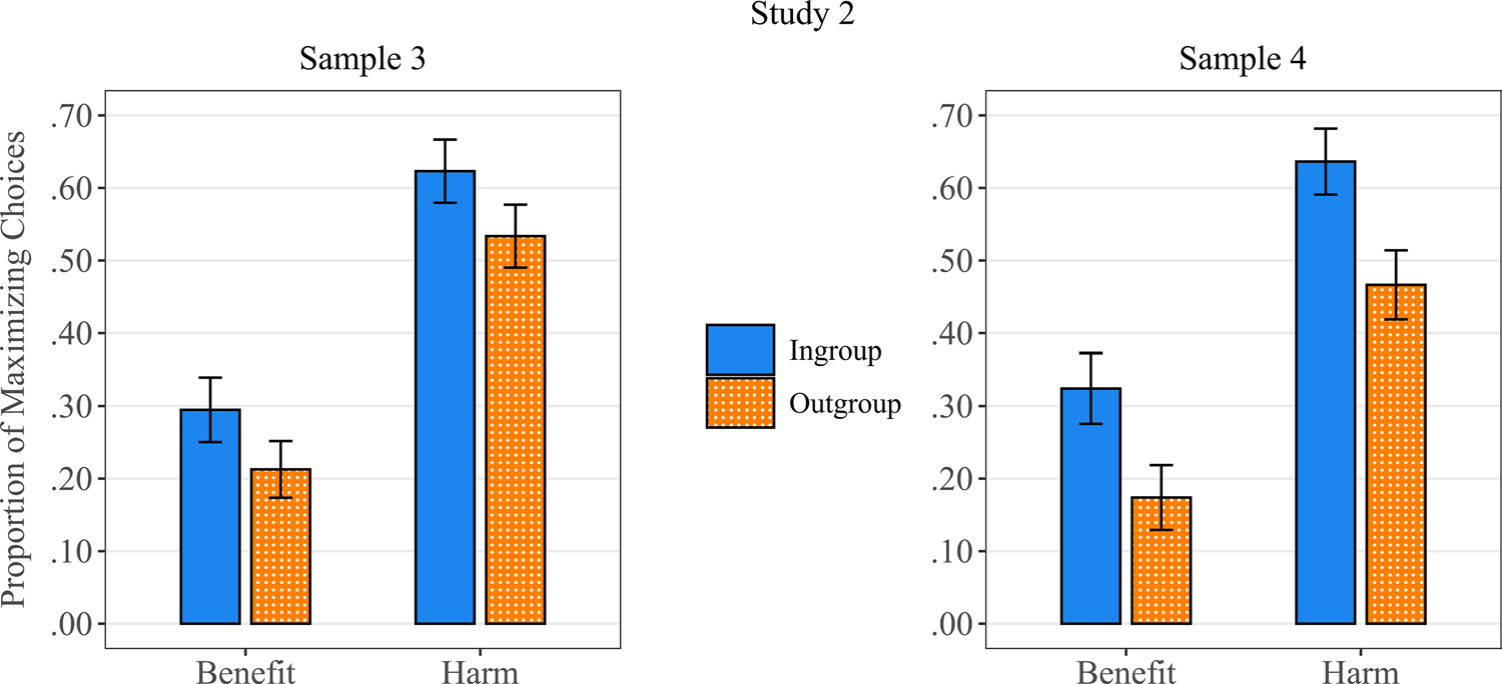
Proportion of outcome Maximizing choices as a function of target group (Ingroup versus Outgroup) and type of outcome (Benefit versus Harm).

Similar to Study 1, in each sample, a 2 × 2 Repeated measures ANOVA was conducted on the proportion of outcome Maximizing choices, with type of outcome (Benefit vs Harm) and group (relative ingroup benefit vs relative outgroup benefit) as within-subject factors (see Fig. 2).

The significant main effect of Outcome type; *F*(1,267) = 120.16, *p* < 0.001, Cohen’s *f* = 1.33, and *F*(1,252) = 98.60, *p* < 0.001, *f* = 1.10, in sample 3 and sample 4 respectively, showed that participants chose the outcome Maximizing option dramatically more often when it served to minimize harm (*M* = 0.58, *SE* = 0.02, and *M* = 0.55, *SE* = 0.03), compared to when it maximized benefit (*M* = 0.25, *SE* = 0.03, and *M* = 0.25, *SE* = 0.02).

The significant main effect of beneficiary group; *F*(1,267) = 23.93, *p* < 0.001, *f* = 0.35, and *F*(1,252) = 56.79, *p* < 0.001, *f* = 0.58, in sample 3 and sample 4 respectively, again demonstrated that participants were also more inclined to choose the Maximizing option when this option was relatively more favorable to the ingroup member compared to the outgroup member (*M* = 0.46, *SE* = 0.02, and *M* = 0.48, *SE* = 0.02), instead of the other way around (*M* = 0.37, *SE* = 0.02, and *M* = 0.32, *SE* = 0.02).

Finally, again, no significant interaction effect was found; *F*(1,267) = 0.62, *p* = 0.803, *f* = 0.02, and *F*(1,252) = 0.32, *p* = 0.570, *f* = 0.04, in sample 3 and sample 4, respectively.

**Figure 3:**
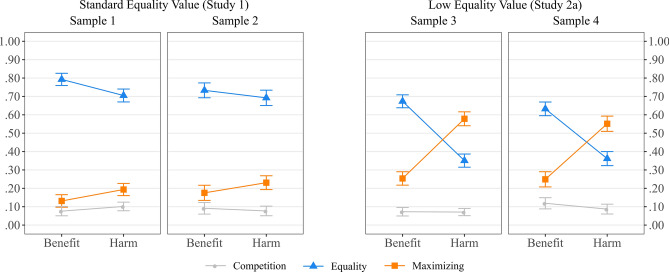
Proportion of Equality, Maximizing, and Competition choices as a function of type of outcome (Benefit versus Harm) and Equality option value (Standard versus Low) across the four samples of Study 1 and Study 2a.

Compared to the samples of Study 1, the increased proportion of outcome Maximizing choices in harm allocation situations is especially remarkable. This is visualized in Fig. 3, which shows that in all the other combinations, Equality choices still represent a vast majority of all choices, but the tables are flipped in the low value equality with harm distribution conditions.

### General distribution of equality, maximizing, and competition choices within groups (Study 2b)

The distribution of choices across the second set of matrices in sample 3 and 4 was computed. Given that this set contains both matrices with standard values and low values for the Equality option, we expected distributions that lie in between those of Study 1 and Study 2a with regard to Equality and Maximizing choices. Also, given that in this second set, the distribution within each matrix does not pertain members from different groups, but instead between ingroup members or between outgroup members, it should make little sense for participants to choose the Competitive option, and hence we expect that the instances in which this option is chosen would be negligible.

The results show no overall, significant difference in sample 3 between the number of times the Equality option (*M* = 0.51, *SE* = 0.02) and the Maximizing option (*M* = 0.46, *SE* = 0.02) was chosen; *t*(267) = 1.13, *p* = 0.261, Cohen’s *d* = 0.14, whereas in sample 4 the Equality option (*M* = 0.59, *SE* = 0.02) is chosen significantly more often than the Maximizing option (*M* = 0.38, *SE* = 0.02); *t*(252) = 4.74, *p* < 0.001, *d* = 0.59. As expected, the Competition option was chosen only very rarely (*M* = 0.03, *SE* = 0.01; *M* = 0.03, *SE* = 0.01), and significantly less often than the Maximizing option; *t*(267) =  − 17.75, and *t*(252) =  − 15.23, both *p* < 0.001, both *d* > 1.42.

### Impact of outcome type, group benefit, and equality value on maximizing choice (Study 2b)

**Figure 4:**
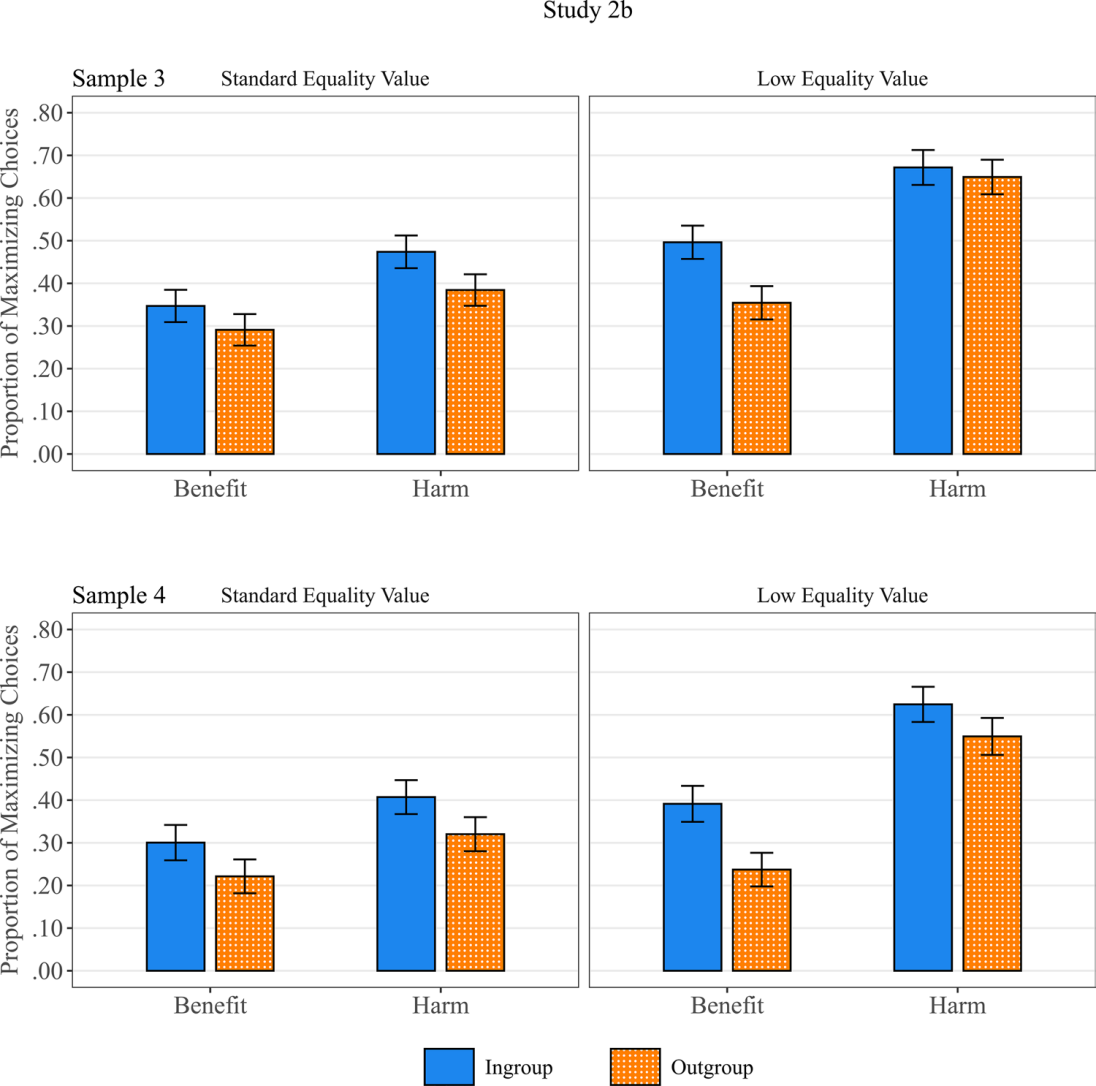
Proportion of Maximizing choices as a function of target group (all Ingroup versus all Outgroup), type of outcome (Benefit versus Harm), and Equality Value (Standards versus Low), for Sample 3 and Sample 4.

We tested whether Maximizing choices are influenced by the type of outcome, the value of the Equality option, and the type of group to which both targets belong. Therefore, in each sample, a 2 × 2 × 2 Repeated measures ANOVA was conducted on the proportion of Maximizing choices, with type of outcome (Benefit vs Harm), group (two ingroup members versus two outgroup members), and value of the Equality option (standards versus low), as within-subject factors (see Fig. 4).

The significant main effect of Outcome type; *F*(1,267) = 74.24, *p* < 0.001, Cohen’s *f* = 1.00, and *F*(1,252) = 101.77, *p* < 0.001, *f* = 1.00, in sample 3 and sample 4 respectively, showed that participants chose the Maximizing option clearly more often when it served to minimize harm (*M* = 0.55, *SE* = 0.03, and *M* = 0.48, *SE* = 0.04), compared to when it maximized benefit (*M* = 0.37, *SE* = 0.03, and *M* = 0.29, *SE* = 0.02).

The significant main effect of target group; *F*(1,267) = 38.71, *p* < 0.001, *f* = 0.45, and *F*(1,252) = 45.18, *p* < 0.001, *f* = 0.53, in sample 3 and sample 4 respectively, demonstrated that participants were also more inclined to choose the Maximizing option when they had to distribute resources between members of the own group (*M* = 0.50, *SE* = 0.02, and *M* = 0.43, *SE* = 0.02), compared to between members of the outgroup (*M* = 0.42, *SE* = 0.02, and *M* = 0.33, *SE* = 0.02).

The significant main effect of Equality value; *F*(1,267) = 95.78, *p* < 0.001, *f* = 0.97, and *F*(1,252) = 59.79, *p* < 0.001, *f* = 0.74, in sample 3 and sample 4 respectively, demonstrated that participants were also more inclined to choose the Maximizing option when the Equality option was objectively low in value (*M* = 0.54, *SE* = 0.02, and *M* = 0.45, *SE* = 0.02), compared to the standard value (*M* = 0.37, *SE* = 0.03, and *M* = 0.31, *SE* = 0.02).

However, one significant interaction effect consistently emerged in both samples, qualifying the latter main effect. In particular, the effect of Equality value was dependent on the type of Outcome, *F*(1,267) = 19.77, *p* < 0.001, *f* = 0.36, and *F*(1,252) = 33.07, *p* < 0.001, *f* = 0.45. As can be seen in Fig. 4, for matrices where penalties had to be allocated, a low value of the Equality option yielded much more Maximizing choices compared to matrices with a standard value for the Equality option, but this effect was substantially reduced for the allocation of benefits. This interaction effect is very much in line with the overview of Study 1 and Study 2a, presented in Fig. 3.

Additionally, whereas no further interaction effects were significant in sample 4, there were two additional interactions in sample 3. Closer inspection of these effects showed that they merely qualified the strength but not the direction of the main effect of Outcome type, and of the interaction between Outcome type and Equality Value. In particular, the impact of harm versus benefit distribution was present for both outgroup and ingroup distribution, but slightly stronger when the allocation only involved outgroup members, *F*(1,267) = 4.22, *p* = 0.04, *f* = 0.12. Similarly, the larger effect of Equality value in harm distribution compared to benefit distribution was significant in both groups but stronger for outgroup member distribution; *F*(1,267) = 13.01, *p* < 0.001, *f* = 0.22.

## General discussion

The present studies aimed to examine utilitarian (i.e., outcome maximizing) moral decision making, going beyond the traditional sacrificial dilemma methodology. By using an adapted version of the allocation task in the minimal group paradigm^22^, our approach not only allowed to pit utilitarianism against common equality-based morality, but also to study these modes of morality for both harm and benefit, and to directly address the role of group identity and impartiality. As such we are able to advance insight in some issues that are at the center of the current debate about the nature, focus, and context of utilitarian morality (see,^6–8,21^). Across the four samples studied, the main results are remarkably consistent, demonstrating that people generally prefer an equal distribution of outcomes over an outcome maximizing distribution in the allocation matrices, but both the outcome-context of the decision, and the group identity of the targets significantly influence how likely people are to make maximizing decisions.

### Minimizing harm versus maximizing benefit: conclusions and implications

In all study variations and samples, participants were significantly more likely to choose the maximizing option when it served to minimize harm compared to when it served to maximize benefit. This outcome-context distinction became especially pronounced in Study 2a, where the adapted equality option in fact represented an unambiguously anti-utilitarian alternative that either minimized overall and individual benefit or maximized overall and individual harm. Indeed, whereas this situation did prompt participants to choose the outcome maximizing option much more frequently when it served to minimize harm, it still did not sway participants to adopt a more maximizing approach when distributing benefits. This clear difference between harm and benefit situations attests to the assertion that moral cognition is indeed inherently more attuned to evaluating actions and decisions that involve harm compared to benefit (see,^16^). In addition to its theoretical importance with respect to the recent call to focus more on the beneficence component of utilitarianism (e.g.,^7,8^), the observation that lay people seem to be especially rigid in their equality-based morality when it comes to distribution of benefits may also be most relevant for recent societal movements that advocate a more utilitarian approach to charity, such as the Effective Altruism movement^32^. Indeed, the present findings suggest that in areas that involve moral reasoning and decisions about how to best distribute benefits to others in need, prompting people to do so in an utilitarian way may be especially challenging when it has to compete with equality-based morality. Although speculative at this time, one potentially fruitful approach might be to frame the message in such a way that it directly appeals to equality-based morality in the public but still achieves utilitarian outcomes. For example, rather than framing a particular initiative as resulting in the most overall benefit (i.e., the most benefit to the highest number of people), it may be framed as resulting in a reduction of the inequality gap for the highest number of people. Although the result (i.e., maximizing overall benefit) would be the same and even the utilitarian logic is identical, the latter framing may more directly appeal to equality-based morality, even if it does not mean that people are treated equally.

### Impartiality: conclusions and implications

A second important consideration based on the current findings pertains to the issue of impartiality in utilitarian decision making. Unlike the traditional sacrificial dilemmas, which feature anonymous targets without any group identity^21^, the minimal group paradigm explicitly incorporates the distinction between an ingroup and an outgroup, even if the content and meaning of this distinction is indeed minimal. Consistently across the samples and designs, we found that although participants rarely chose the competition option that blatantly favored the ingroup member at the expense of the outgroup member, they were remarkably more inclined to choose the maximizing option if it was most beneficial for the ingroup member compared to when it provided a relative advantage to the outgroup member. This effect occurred for both distributions of benefits and harms. In fact, on average across all conditions and samples, maximizing options that favored the ingroup member were chosen almost twice as frequently as maximizing options that relatively favored the outgroup member. This finding suggests that for a considerable number of participants who chose the maximizing option with ingroup advantage, it may have been used as a “soft” and rationally more defensible version of the competition option, rather than reflecting a true “impartial utilitarian” mindset. Arguably, only a choice for the maximizing option that relatively favored the outgroup member can be considered as reflecting genuinely impartial utilitarian morality. Combined with the lower consideration for the maximizing alternative when distributing benefits (compared to harms), behavioral displays of what has been called ‘impartial beneficence’^6^ were relatively rare in our experiments using a minimal group paradigm, and hence one could speculate that such choices are probably also uncommon in other situations where people have to distribute benefits. The apparent greater concern to maximize overall outcomes when it simultaneously serves ingroup interests also emerged when allocations had to be made within groups. Indeed, in Study 2b, participants made more maximizing decisions when allocating resources between two members of the ingroup compared to between two members of the outgroup. This further attests to the conclusion that people are often partial in their seemingly ‘utilitarian’ morality, but also indicates that advocating a more utilitarian approach to, for example, charity may have greater chances of success when it targets people within one’s own community, compared to targets within other communities, or across communities.

## Conclusion

Taking the study of utilitarianism out of the traditionally used sacrificial dilemma methodology, we demonstrate that lay people generally are not very inclined to display utilitarian morality in their decision making when it has to compete with equality-based morality. Only when equal distribution options also maximize overall harm (but not if they minimize overall benefit), do people show a considerable shift towards decisions that maximize the overall outcome. Moreover, such maximizing decisions are more likely if they also serve the ingroup compared to when they rather serve the outgroup. These findings have meaningful theoretical and practical implications. Initiatives that aim to advance the use of impartial utilitarianism in guiding decision making, such as the Effective Altruism initiative^32^, have often met with resistance and negative reception in parts of the general public, which is commonly attributed to its controversial messages about instrumental harm (see,^6,7^). However, the present findings suggest that although the proposed solution to put a stronger focus on impartial beneficence may face less fierce (emotional) resistance, it may not necessarily lead to more impartial utilitarian behavior in domains like charity. Indeed, even when being pro-social, most people appear to rigidly adhere to equality principles especially to allocate benefits, and in cases where they do take an seemingly utilitarian approach, it is often not impartial.

## Materials and methods

Specific materials, as well as all data, and additional analyses are available through the osf page at https://osf.io/mbcqp/.

The experimental protocols were approved by the Ethical Committee of the Faculty of Psychology and Educational Sciences of Ghent University as part of a larger project (ref: 2016/86 Amended), and all methods were carried out in accordance with the relevant guidelines and regulations. All participants took part in the study voluntarily and informed consent was obtained from all subjects and/or legal guardian for minors (participant minimum age: 17 years).

### Study 1

#### Participants

Data were collected in two independent samples of undergraduate students: Sample 1 with *N* = 217, and Sample 2 with *N* = 208. Sample size was determined by the availability of subjects. A power sensitivity test demonstrated that both samples have at least 80% power to test for small to medium sized within-subjects main effects of Cohen’s *f* ≥ 0.195. Participants completed the study either online on their home computer (Sample 1; 90.8% women, *M*age = 20.12, *SD* = 3.61), or as part of an experimental session in medium-sized groups on desktop computers in the lab (Sample 2; 78.4% women, *M*age = 18.79, *SD* = 2.94).

#### Procedure

Participants were presented with two paintings; one by Kandinsky (painting A) and one by Klee (painting B). After choosing which painting they liked best, they received a message reading: “Congratulations, you chose painting A (B). You are now a member of group Kandinsky (Klee)”. Next they received instructions that their next task would be to allocate points to pairs of other students, identified only by their participant code and the group to which they belonged based on their painting preference earlier. They were informed that these students (but not themselves) would have to come to the lab later to do a task, which would have a variable difficulty and duration: the number of points they allocated to a particular student would determine the difficulty and duration for that particular student (the more points, the easier and shorter the task).

#### Measures and materials

Participants were presented with a series of six matrices, which were modelled after Tajfel’s matrices, but were substantially simplified to serve the present study’s objectives. In particular, we wanted to make sure that participants were able to easily and immediately understand the idea behind each option. Therefore, for each matrix, there were only 3 options in a variable order: Competition, Equality, and Maximizing option (see above). The matrices presented either a distribution of bonus points^2^, penalties^2^, or a combination thereof^2^. The combination matrices were primarily filler items added for exploratory purposes and were not included in the main analyses, but additional analyses with these combination matrices are presented at the osf page. An example matrix (distribution of bonus points with utilitarian ingroup member benefit) asked participants who chose Kandinsky to distribute points between KANDINSKY group member dsf-042 and KLEE group member jkz-128, by choosing one of the following three options: + 6/+ 4 vs + 4/+ 4 vs + 7/+ 2 (Maximizing, Equality, and Competition, respectively). Additionally, participants completed measures of Right-Wing Authoritarianism^33^, Social Dominance Orientation^34^, and the Oxford Utilitarianism Scale^6^. Given that effects of the individual differences measures were very limited compared to the experimental effects, analyses with these variables are not discussed but can be found in full in the osf page.

### Study 2

#### Participants

Data were collected in two new, independent samples of undergraduate students: Sample 3 with *N* = 268, and Sample 4 with *N* = 253. Sample size was determined by the availability of participants. A power sensitivity test demonstrated that each sample has at least 80% power to test for small to medium sized within-subjects main effects of Cohen’s *f* ≥ 0.177. All participants completed the study online, either on their home computer or in medium-sized groups on desktop computers in the lab. The samples consisted of 84.3% and 70.8% women and the mean age was 20.58 (*SD* = 4.11) and 18.70 (*SD* = 1.81) years.

#### Procedure

Participants were again presented with two paintings. However, as a small adaptation to Study 1, the presented paintings were of unknown artists and were presented as the work of two fictitious artists (Dusek versus Tausig, see^35^). After choosing a painting, all participants were told they had chosen the painting of Dusek (to simplify the dataset). In all other respects, the procedure for the first part (Study 2a) was identical to Study 1. In the second part (Study 2b), we presented participants with an additional set of 8 matrices that contained both stimuli with a standard Equality value and with a low Equality value. Unlike the previous set however, in these matrices, participants had to distribute the bonuses/penalties between either two ingroup members, or between two outgroup members.

#### Measures and materials

Similar to Study 1, for Study 2a, participants were again presented with 6 matrices. However, the objective value of the Equality option was considerably reduced compared to Study 1, by making this the option that objectively left everyone worse off than the other options. In particular, in the Equal distribution option, benefits were equal for the two targets (e.g. + 2/+ 2), but presented the lowest overall benefit, as well as the lowest benefit for the individuals when compared to the other options (e.g., + 6/+ 4 and + 7/+ 2). For Study 2b, participants received an additional 8 matrices (harm or benefit only). Unlike the previous set however, in these matrices, participants had to distribute the bonuses/penalties between either two ingroup members, or between two outgroup members.
